# Prognostics of Lithium-Ion Batteries Based on Wavelet Denoising and DE-RVM

**DOI:** 10.1155/2015/918305

**Published:** 2015-08-30

**Authors:** Chaolong Zhang, Yigang He, Lifeng Yuan, Sheng Xiang, Jinping Wang

**Affiliations:** ^1^School of Electrical Engineering and Automation, Hefei University of Technology, Hefei 230009, China; ^2^School of Physics and Electronic Engineering, Anqing Normal University, Anqing 246011, China

## Abstract

Lithium-ion batteries are widely used in many electronic systems. Therefore, it is significantly important to estimate the lithium-ion battery's remaining useful life (RUL), yet very difficult. One important reason is that the measured battery capacity data are often subject to the different levels of noise pollution. In this paper, a novel battery capacity prognostics approach is presented to estimate the RUL of lithium-ion batteries. Wavelet denoising is performed with different thresholds in order to weaken the strong noise and remove the weak noise. Relevance vector machine (RVM) improved by differential evolution (DE) algorithm is utilized to estimate the battery RUL based on the denoised data. An experiment including battery 5 capacity prognostics case and battery 18 capacity prognostics case is conducted and validated that the proposed approach can predict the trend of battery capacity trajectory closely and estimate the battery RUL accurately.

## 1. Introduction

Lithium-ion batteries have been widely used as crucial components and important backup elements for many systems including electric vehicles, consumer electronics, and aerospace electronics. Compared with other kind of batteries, the lithium-ion battery has advantages of high power density, high galvanic potential, light weight, and long cycle life. However, irreversible chemical and physical changes take place in the lithium-ion battery with usage and aging. As a result, the battery health degrades gradually until it is no longer usable eventually. The consequence of the battery failure would lead to the capacity degradation, operation loss, downtime, and even catastrophic failure. Hence, prognostics and health management (PHM) of the lithium-ion battery has been an active field which has attracted an increasing attention today [1–12].

PHM is an enabling discipline composed of technologies and approaches to estimate the reliability of an application system in its actual life cycle conditions to provide ample forewarning before a failure occurs and mitigates system risk. PHM of the lithium-ion battery includes evaluating its state of health (SOH) and predicting its remaining useful life (RUL). Meanwhile, the gradual decreased capacity of the battery is a universally used SOH indicator that can track its health degradation.

Model-based and data-driven approaches are two main kinds of approaches to the battery capacity prognostics. Model-based approaches employ mathematical representations to character the understanding of the battery failure and underlying the battery capacity's degradation model. Extended Kalman filtering (EKF) [1, 2], nonlinear model [3, 4], and particle filtering (PF) [5, 6] are commonly used model-based methods for the battery capacity estimation. However, an accurately analytical and universally accepted model to track the battery capacity degradation and evaluate the battery RUL is usually difficult to be derived because of the complex electronic system, noise, data availability, uncertain environments, and application constraints. Data-driven approaches utilize statistical and machine learning techniques to evaluate the battery capacity and predict the battery RUL. The approaches avoid constructing complex physical models and have been applied in many relative works [7–12]. Artificial neural network is a widely used data-driven approach to the battery capacity prognostics [7, 8]. However, it has disadvantages of poor generalization, difficult structure confirmation, and low convergence rate. Support vector machine (SVM) is a machine learning tool [13] characterized by the usage of kernel functions and it has been utilized to estimate the battery RUL [9, 10]. Relevance vector machine (RVM) is a Bayesian sparse kernel technique [14] with usage of much fewer kernel functions and higher performance compared to the SVM. Meanwhile, RVM has been applied to the research field [11, 12].

The measured battery capacity data are often subject to the different levels of noise pollution because of the impact of disturbances, measurement errors, stochastic load, and other unknown behaviors in batteries. Capacity prognostics based on the noisy data cannot produce accurate predict results. Therefore, it is significantly important to preprocess the measured capacity data for the purpose of extracting the original data and removing the noise. To address the problem and estimate the battery RUL accurately, a novel battery capacity prognostics approach is presented in the paper. A wavelet denoising method performed with different thresholds is employed to process the measured data to reduce the uncertainty and extract the useful information. RVM optimized by differential evolution (DE) algorithm is utilized to estimate the battery RUL. An experiment including battery 5 capacity prognostics case and battery 18 capacity prognostics case is conducted, which validates that the proposed approach can predict the trend of battery capacity trajectory closely and estimate the RUL accurately.

The material in the paper is organized in the following order: Section 2 describes the strategy of wavelet denoising method.  Section 3 introduces RVM algorithm and its parameter optimization by using DE algorithm.  Section 4 illustrates the experiment procedure, presents the experiment results, and gives discussions. Finally, conclusions are drawn in Section 5.

## 2. Wavelet Denoising

The measured capacity data of batteries often suffer from the different levels of noise pollution. Experiment with noisy data cannot yield the accurate RUL. As a result, it is very important to preprocess the capacity data for the purpose of extracting the original data. Wavelet denoising method is adopted to address the concern.

Assume that the measured capacity data capacity(*c*) is comprised by

(1) $$\begin{array}{c} \text{capacity}\left(c\right)=x\left(c\right)+\sigma \left(c\right), \end{array}$$

where *x*(*c*) is the original data; *σ*(*c*) is the additive noise; and *c* refers to the cycle which is a time index.

Assume that *Z* is an integers set, {*V*_*t*_}_*t*∈*Z*_ is an orthogonal multiresolution analysis, and {*W*_*t*_}_*t*∈*Z*_ is the associated wavelet space. The capacity(*c*) projection on *V*_*t*_ is

(2) $$\begin{array}{c} P_{V_{t}}=P_{V_{t+1}}+P_{W_{t+1}}=\underset{i\in Z}{\sum }c_{t+1}^{i}\phi_{t+1,i}+\underset{i\in Z}{\sum }d_{t+1}^{i}\psi_{t+1,i}, \end{array}$$

where *P*_*V*_*t*+1__ and *P*_*W*_*t*+1__ denote the capacity(*c*) projections on *V*_*t*+1_ and *W*_*t*+1_ at 2^*t*+1^ resolution, respectively; *c*_*t*+1_^*i*^ and *d*_*t*+1_^*i*^ refer to the scaling coefficient and wavelet coefficient of capacity(*c*) at 2^*t*+1^ resolution, respectively; *ϕ*_*t*+1_ and *ψ*_*t*+1_ represent the scaling function and wavelet function of capacity(*c*) at 2^*t*+1^ resolution, respectively. Therefore, *c*_*t*+1_ and *d*_*t*+1_ characterize the approximations and details of capacity(*c*) at 2^*t*+1^ resolution, respectively. Correspondingly, {*V*_*t*_}_*t*∈*Z*_ can be decomposed as

(3) $$\begin{array}{c} V_{t}=W_{t+1}⊕V_{t+1}=W_{t+1}⊕\left(W_{t+2}⊕V_{t+2}\right)=W_{t+1}⊕W_{t+2}⊕\left(W_{t+3}⊕V_{t+3}\right)=W_{t+1}⊕W_{t+2}⊕W_{t+3}⊕\cdots . \end{array}$$

By using the multilevel wavelet decomposition, discrete approximation coefficients and detail coefficients are produced. The detail coefficients with small absolute values are considered to be noise. Generally, the traditional wavelet denoising method is setting the detail coefficients below a threshold to zero and reconstructing the denoised data by using the rest coefficients. Sqtwolog threshold, rigorous threshold, heursure threshold, and minimax threshold are commonly used rules to yield the threshold. In the work, the wavelet denoising is performed twice with sqtwolog threshold rule and minimax threshold rule, respectively.

The sqtwolog threshold rule produces the threshold which can yield good performance multiplied by a small factor proportional to log(length(capacity)):

(4) $$\begin{array}{c} {\text{Threshold}}_{\text{sqtwolog}}=\sqrt{2\log n}, \end{array}$$

where *n* is the length of the capacity set.

The minimax threshold rule brings about the minimum of the maximum mean square error generated for the worst function with a given set by using the minimax principle. The threshold is defined as

(5) $$\begin{array}{c} {\text{Threshold}}_{\text{minimax}}=a+b*\frac{\log\left(n\right)}{\log\left(2\right)}, \end{array}$$

where *a* and *b* are factors which are generally set to 0.3936 and 0.1829, respectively.

The minimax threshold is obviously lower than the sqtwolog threshold in magnitude with a signal. Wavelet denoising with the sqtwolog threshold can weaken the strong noise obviously. Meanwhile, wavelet denoising with the minimax threshold can remove the weak noise effectively. The wavelet denoising strategy in the work is implementing wavelet denoising with the sqtwolog threshold firstly and then performing wavelet denoising with the minimax threshold.

## 3. DE-RVM

### 3.1. RVM

RVM is firstly presented in [14] and has generated demonstrative effect in prognostics [15–17]. The algorithm is a Bayesian treatment which provides probabilistic interpretation of the output. The relevance vectors and weights are obtained by maximizing a marginal likelihood.

Assume that {**x**_*i*_, *t*_*i*_}_*i*=1_^*N*^ is the input data. The target *t*_*i*_ is obtained by

(6) $$\begin{array}{c} t_{i}=y\left(x_{i};w\right)+\varepsilon_{i}, \end{array}$$

where **w** = (*w*_0_, *w*_1_,…, *w*_*N*_)^*T*^ and *ε*_*i*_ is the noise with mean zero and variance *σ* ^2^.

Assume that *t*_*i*_ is independent and the likelihood of complete dataset can be defined as

(7) $$\begin{array}{c} p\left(t\;|\;w,\sigma^{2}\right)={\left(2\pi \sigma^{2}\right)}^{-N/2}\exp\left\{-\frac{1}{2\sigma^{2}}{\left\|t-\varphi w\right\|}^{2}\right\}, \end{array}$$

where **t** = (*t*_1_, *t*_2_,…, *t*_*N*_)^*T*^ and **φ** is a *N* × (*N* + 1) design matrix with **φ** = [*φ*(**x**_1_), *φ*(**x**_2_),…, *φ*(**x**_*N*_)]^*T*^ and *φ*(**x**_*i*_) = [1, *K*(**x**_*i*_, **x**_1_), *K*(**x**_*i*_, **x**_2_),…, *K*(**x**_*i*_, **x**_*N*_)]^*T*^.

Maximum likelihood estimations of **w** and *σ* ^2^ in (7) often result in overfitting. Hence, an explicit zero-mean Gaussian prior probability distribution is defined in order to constrain the parameters as

(8) $$\begin{array}{c} p\left(w\;|\;\alpha \right)=\overset{N}{\underset{i=0}{\prod }}N\left(w_{i}\;|\;0,\alpha_{i}^{-1}\right), \end{array}$$

where **α** is a *N* + 1 hyperparameters vector.

Using Bayes' rule, the posterior probability about all of the unknown parameters can be obtained by

(9) $$\begin{array}{c} p\left(w,\alpha ,\sigma^{2}\;|\;t\right)=\frac{p\left(t\;|\;w,\alpha ,\sigma^{2}\right)p\left(w,\alpha ,\sigma^{2}\right)}{\int p\left(t\;|\;w,\alpha ,\sigma^{2}\right)p\left(w,\alpha ,\sigma^{2}\right)dw\;d\alpha \;d\sigma^{2}}. \end{array}$$

However, the normalizing integral ∫*p*(**t**∣**w**, **α**, *σ* ^2^)*p*(**w**, **α**, *σ* ^2^)*d* **w** *d* **α** *dσ* ^2^ cannot be easily executed. Therefore, *p*(**w**, **α**, *σ* ^2^∣**t**) can be instead decomposed as

(10) $$\begin{array}{c} p\left(w,\alpha ,\sigma^{2}\;|\;t\right)=p\left(w\;|\;t,\alpha ,\sigma^{2}\right)p\left(\alpha ,\sigma^{2}\;|\;t\right). \end{array}$$

Based on the Bayes' rule, the posterior distribution of weights is obtained through

(11) $$\begin{array}{c} p\left(w\;|\;t,\alpha ,\sigma^{2}\right)=\frac{p\left(t\;|\;w,\sigma^{2}\right)p\left(w\;|\;\alpha \right)}{p\left(t\;|\;\alpha ,\sigma^{2}\right)},\\ p\left(w\;|\;t,\alpha ,\sigma^{2}\right)={\left(2\pi \right)}^{-\left(N+1\right)/2}{\left|\Sigma \right|}^{-1/2}\exp\left\{-\frac{1}{2}{\left(w-\mu \right)}^{T}\Sigma^{-1}\left(w-\mu \right)\right\}, \end{array}$$

where the posterior mean and covariance are

(12) $$\begin{array}{c} \mu =\sigma^{-2}\Sigma \varphi^{T}t,\\ \Sigma ={\left(\sigma^{-2}\varphi^{T}\varphi +A\right)}^{-1}, \end{array}$$

where *A* = diag(*α*_0_, *α*_1_,…, *α*_*N*_).

Because of the uniform hyperpriors, *p*(**t**∣**α**, *σ* ^2^) is described by

(13) $$\begin{array}{c} p\left(t\;|\;\alpha ,\sigma^{2}\right)=\int p\left(t\;|\;w,\sigma^{2}\right)p\left(w\;|\;\alpha \right)dw\\ ={\left(2\pi \right)}^{-N/2}{\left|\sigma^{2}I+\varphi A^{-1}\varphi^{T}\right|}^{-1/2}\exp\left\{-\frac{1}{2}t^{T}{\left(\sigma^{2}I+\varphi A^{-1}\varphi^{T}\right)}^{-1}t\right\}. \end{array}$$

The maximum posterior (MP) estimate of the weights is described by the posterior mean, which depends on the value of **α** and *σ* ^2^. The estimates of **α**_MP_ and *σ*_MP_^2^ are acquired by maximizing the marginal likelihood. Tipping [14] presents the iterative formulas for **α**_MP_ and *σ*_MP_^2^ as

(14) $$\begin{array}{c} \alpha_{i}^{new}=\frac{1-\alpha_{i}\Sigma_{ii}}{\mu_{i}^{2}},\\ {\left(\sigma^{2}\right)}^{new}=\frac{{\left\|t-\varphi \mu \right\|}^{2}}{N-\sum_{i}\left(1-\alpha_{i}\Sigma_{ii}\right)}, \end{array}$$

where Σ_*ii*_ is the *i*th diagonal element of the posterior weight covariance.

Assume that **x**_*∗*_ is a new input and the probability distribution of the output *t*_*∗*_ is obtained by

(15) $$\begin{array}{c} p\left(t_{*}\;|\;t,\alpha_{MP},\sigma_{MP}^{2}\right)=\int p\left(t_{*}\;|\;w,\sigma_{MP}^{2}\right)p\left(w\;|\;t,\alpha_{MP},\sigma_{MP}^{2}\right)dw. \end{array}$$

It can be easily obtained for both integrated terms are Gaussian, and the result is also a Gaussian form

(16) $$\begin{array}{c} p\left(t_{*}\;|\;t,\alpha_{MP},\sigma_{MP}^{2}\right)=N\left(t_{*}\;|\;y_{*},\sigma_{*}^{2}\right). \end{array}$$

The mean and the variance are

(17) $$\begin{array}{c} y_{*}=\mu^{T}\varphi \left(x_{*}\right),\\ \sigma_{*}^{2}=\sigma_{MP}^{2}+\varphi {\left(x_{*}\right)}^{T}\Sigma \varphi \left(x_{*}\right). \end{array}$$

Gaussian radial basis function is selected as the kernel function for its powerful nonlinear processing capability, and the function is defined as

(18) $$\begin{array}{c} K\left(x,x_{i}\right)=\exp\left[-\frac{{\left(x-x_{i}\right)}^{2}}{2\gamma^{2}}\right], \end{array}$$

where *γ* is the width factor which needs to be predetermined for it is crucially important to the predict performance.

### 3.2. DE Algorithm

DE algorithm is a population-based and stochastic search approach [18] and has shown superior performance on nonlinear, nonconvex, and nondifferentiable optimization problems [19–21]. DE algorithm starts with an initial population vector, which is randomly generated in a solution space. Assume that *N* is the population size and *X*_*ri*,*G*_  (*i* = 1,2,…, *N*) is a solution vector of the generation *G*. For the classical DE algorithm, mutation and crossover are utilized to generate trial vectors, and selection is used to select the better vectors.

*Mutation*. For each vector *X*_*ri*,*G*_, a mutant vector *V*_*i*,*G*_ is generated by

(19) $$\begin{array}{c} V_{i,G}=X_{r1,G}+F\left(X_{r2,G}-X_{r3,G}\right)\;r1\neq r2\neq r3\neq i, \end{array}$$

where *r*1, *r*2, and *r*3 are random integer indexes selected from {1,2,…, *N*}; *F* is the scale fact which determines the amplification of the difference vector (*X*_*r*2,*G*_ − *X*_*r*3,*G*_), and *F* ∈ [0,2]. 

*Crossover*. The crossover operation refers to yielding the trial vector by using the mutant vector *V*_*i*,*G*_ and target vector *X*_*i*,*G*_:

(20) $$\begin{array}{c} U_{ji,G}=\left\{\begin{array}{cc} V_{ji,G}, & {rand}_{j}\leq C_{r}\text{ or }j=k\\ X_{ji,G}, & \text{otherwise,} \end{array}\right. \end{array}$$

where *j* = 1,2,…, *D*, and *D* is the problem dimension; *C*_*r*_ ∈ [0,1] is the predefined crossover constant; rand_*j*_ is the *j*th evaluation which is randomly generated between 0 and 1; *k* ∈ {1,2,…, *D*} and it is a random index. 

*Selection*. Assume that *f*(*x*) is a minimization problem. The greedy selection scheme is defined as

(21) $$\begin{array}{c} X_{i,G+1}=\left\{\begin{array}{cc} U_{i,G}, & \text{if}\;\;f\left(U_{i,G}\right)<f\left(X_{i,G}\right)\\ X_{i,G}, & \text{otherwise.} \end{array}\right. \end{array}$$

The above three steps are repeated until reaching the terminal condition. Then the best vector with minimum fitness value is exported as the result.

### 3.3. Steps of Optimization

DE-RVM refers to the RVM with width factor optimized by DE algorithm. Mean square error (MSE) is used as the fitness function:

(22) $$\begin{array}{c} \text{MSE}=\frac{\sum_{\delta =1}^{H}{\left[z^{*}\left(\delta \right)-z\left(\delta \right)\right]}^{2}}{H}, \end{array}$$

where MSE represents the deviate degree of the predicted data and the original data; *δ* = 1,2,…, *H*, and *H* is the length of the original data; *z*(*δ*) and *z* ^*∗*^(*δ*) are the original data and predicted data, respectively.

The optimization target is to minimize the MSE value, and the optimizing steps are described as follows:

1. Initialize the DE algorithm parameters, which include the population size, scale factor, crossover rate, and maximum generation.
2. Produce the mutant vector and trial vector according to (19) and (20).
3. Determine the next generation vector according to (21).
4. Repeat steps (2) and (3) until the terminated criterion is met.
5. Output the optimized value to the RVM and exit the program.

## 4. Prognostics Experiment 

### 4.1. Experiment Data

**Figure 1:**
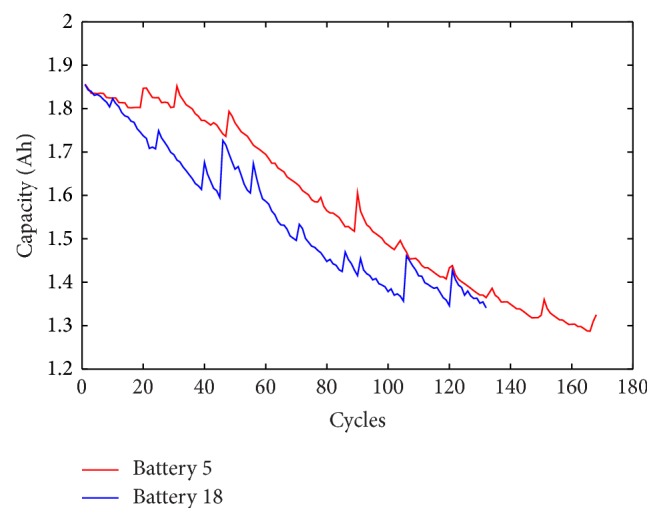
Measured capacity data of batteries.

An experiment is conducted to demonstrate the proposed capacity prognostics approach, and the data were obtained from data repository of NASA Ames Prognostics Center of Excellence [22]. In the data collected procedure, lithium-ion batteries were working under three different operational profiles: charge, discharge, and impedance with a temperature of 25°C. Charging was performed at a 1.5 A constant current until the battery voltage reached 4.2 V and then maintaining the 4.2 V constant voltage until the current dropped to 20 mA. Discharging was running at a 2 A constant current until the battery voltage felled to 2.7 and 2.5 V, which were corresponding to batteries 5 and 18, respectively. Impedance measurement was implemented with an electrochemical impedance spectroscopy frequency sweep ranging from 0.1 Hz to 5 kHz. Repeated charge and discharge cycles led to the accelerated aging of batteries while impedance measurements discovered the changes of the internal battery parameters with aging progresses. The experiments were terminated when the capacity of batteries reached its end-of-life (EOL) threshold, which was about 70% rated capacity. In the experiments, each nominal capacity of lithium-ion battery is 2 Ah and the EOL threshold is set to 1.38 Ah. The lithium-ion batteries 5 and 18 capacity data are shown in Figure 1. It can be observed that the capacity generally degrades with usage for the reason of irreversible physical and chemical changes and at some cycle increases rapidly and shortly due to the impact of disturbances, measurement errors, stochastic load, or other unknown behaviors in the batteries. The length of batteries 5 and 18 capacity data are 166 cycles and 132 cycles, respectively. Meanwhile, their actual cycle lives are 129 and 114, respectively.

### 4.2. Experiment Procedure

**Figure 2:**
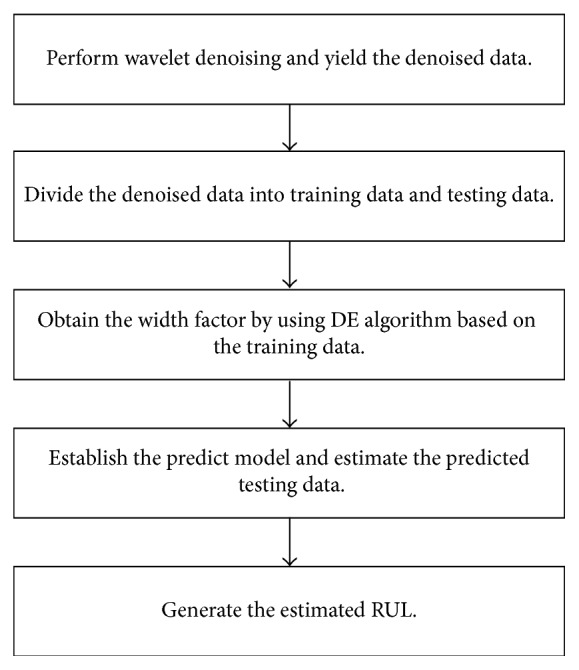
Flowchart of predict steps.

The experiment includes a battery 5 capacity prognostics case and a battery 18 capacity prognostics case. The detailed predict steps of each case are shown in Figure 2 and described as follows:

1. Perform wavelet denoising based on the measured data and obtain the denoised data.
2. Separate the denoised data into training data and testing data. The lengths of the training data in the two battery cases are set to 80 and 70, respectively. Therefore, the lengths of the testing data in two cases are 88 and 62, respectively.
3. By using DE algorithm, a width factor is generated based on the training data.
4. A predict model is constructed by RVM with adopting the optimized width factor, and the predicted testing data are estimated.
5. Generate the estimated RUL.

### 4.3. Experiment Results and Analysis

**Figure 3:**
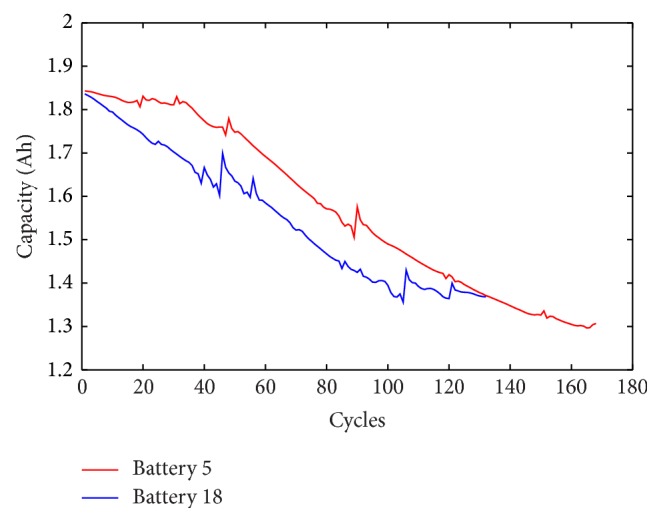
The batteries' capacity data wavelet denoised with the sqtwolog threshold.

**Figure 4:**
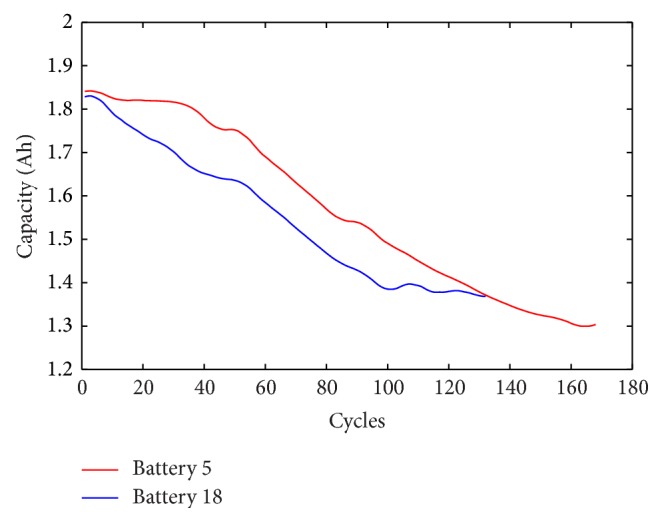
The batteries' capacity data wavelet denoised with the minimax threshold further.

Wavelet denoising implemented twice with different thresholds is employed to process the measured capacity data.  Figure 3 displays the capacity data wavelet denoised with the sqtwolog threshold, and strong peak pulses are weakened obviously compared to Figure 1. Then the denoised capacity data are processed by using wavelet denoising with the minimax threshold to remove the weak noise. The denoised capacity data are shown in Figure 4 and the trajectory of the denoised data is continuous and smooth.

**Figure 5:**
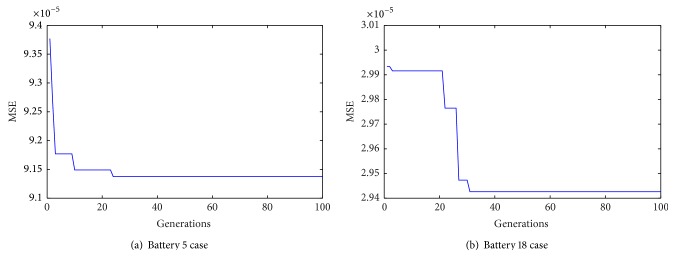
The width factor optimization procedures.

The DE algorithm population size and maximum generation are set to 30 and 100, respectively; *F* is equal to 0.6; *C*_*r*_ is linearly reduced from 0.9 to 0.3.  Figure 5 shows the width factor optimization procedures by using DE algorithm based on batteries 5 and 18 training data, respectively. The corresponding optimized wider factors are 0.5009 and 0.2778 in the two battery cases, respectively.

Adopting the optimized width factor, RVM is used to perform battery capacity prognostics. In order to quantify the prognostic performance, absolute error (AE), and MSE between the original testing data and the predicted testing data, relative accuracy (RA) and *α* − *λ*  accuracy [23] are employed as the measure metrics. The *α* − *λ*  accuracy is applied to verify whether the estimated RUL is within the confidence interval defined by *α*. The metrics are defined as

(23) $$\begin{array}{c} \text{AE}=\text{RU}{\text{L}}_{\text{es}}-\text{RUL},\\ \text{RA}=1-\frac{\left|\text{RU}{\text{L}}_{\text{es}}-\text{RUL}\right|}{\text{RUL}},\\ \alpha -\lambda \text{ accuracy}=\left\{\begin{array}{cc} \text{Yes} & \text{if }{\text{RUL}}_{\text{es}}\in \left[C_{1},C_{2}\right]\\ \text{No} & \text{if others}, \end{array}\right. \end{array}$$

where RUL_es_ refers to the estimated RUL and RUL denotes the actual RUL; *C*_1_ and *C*_2_ are confidence intervals which equal RUL*∗*(1 − *α*) and RUL*∗*(1 + *α*), respectively; *α* is a bound which is set to 0.1 in the experiment.

**Figure 6:**
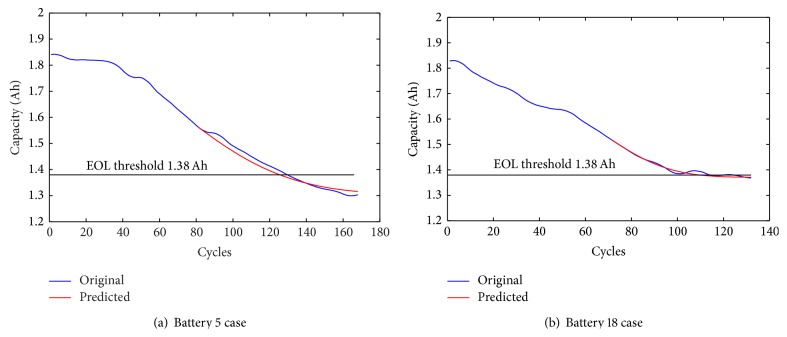
Prediction results.

Actual RULs in the two battery cases are 49 and 44, respectively. The predictions are displayed in Figure 6. Estimated RULs, actual RULs, AEs, RAs, MSEs, and *α* − *λ*  accuracy of two cases are shown in Table 1. As can be seen from Figure 6, the DE-RVM predicts the trend of capacity degradation trajectories of the two cases successfully. Meanwhile this can also be verified by the MSEs in Table 1, which are pretty low in the two cases and this denotes that the predicted testing data are close to the original testing data. RAs are all beyond 90% in two cases which implies high prediction accuracies produced by the DE-RVM. Meanwhile, the estimated RULs in the two cases are both within the confidence interval as the last row shows.

For the purpose of validating the predict performance of the presented prognostics approach, the DE-RVM approach is compared with ANN optimized by DE algorithm (DE-ANN) [24] approach and SVM improved by DE algorithm (DE-SVM) [25] approach. The denoised data of batteries 5 and 18 are used as experiment data. The assessment index adopts RA and MSE. In order to avoid accidental accident in the experiment, each approach is run 10 times and mean results are shown in Table 2. As can be seen from the table, the DE-RVM provides smaller MSE than the DE-ANN and DE-SVM which implies that the data predicted by the DE-RVM are more close to the original data. Meanwhile, the DE-RVM yields higher RA than the DE-ANN and the DE-SVM which characterizes that the DE-RVM can output more accurate prediction than the other two approaches. It can be concluded that the DE-RVM approach significantly outperforms the DE-ANN approach and the DE-SVM approach on the problem of the battery capacity prognostics.

## 5. Conclusions

The gradual decreased capacity of lithium-ion batteries has been used as the SOH indicator in the work. For the reason of the measured battery capacity data often suffering from the different levels of noise pollution, a wavelet denoising method with different thresholds has been presented to generate the denoised data.

The RVM with its width factor optimized by DE algorithm has been used for battery capacity prognostics. Two battery case results have validated that the approach can predict the trend of capacity degradation trajectory closely and estimate the battery RUL accurately. Meanwhile an extend experiment has demonstrated that the proposed DE-RVM approach has higher predict accuracy than the referenced approaches in the battery capacity prognostics.

## Figures and Tables

**Table 1:** Actual RULs, estimated RULs, AEs, RAs, and MSEs of two cases.

Case	Estimated RUL	Actual RUL	AE	RA	MSE	*α* − *λ* accuracy
Battery 5	45	49	−4	91.8%	1.4178*e* − 04	Yes
Battery 18	40	44	−4	90.9%	3.9249*e* − 05	Yes

**Table 2:** RAs and MSEs of the referenced approaches.

Case	DE-ANN		DE-SVM	
	RA	MSE	RA	MSE
Battery 5	85.7%	2.7684*e* − 04	87.8%	2.1507*e* − 04
Battery 18	81.8%	7.3153*e* − 05	86.4%	5.8236*e* − 05
